# Bacterial disease induced changes in fungal communities of olive tree twigs depend on host genotype

**DOI:** 10.1038/s41598-019-42391-8

**Published:** 2019-04-10

**Authors:** Teresa Gomes, José Alberto Pereira, Teresa Lino-Neto, Alison E. Bennett, Paula Baptista

**Affiliations:** 1 CIMO/Instituto Politécnico de Bragança, Campus de Santa Apolónia, 5300-253 Bragança, Portugal; 2 Biosystems & Integrative Sciences Institute (BioISI), Plant Functional Biology Center (CBFP), University of Minho, Campus de Gualtar, 4710-057 Braga, Portugal; 3 Dept of Evolution, Ecology & Organismal Biology, The Ohio State University, 318 W. 12th Ave., 300 Aronoff Laboratory, Columbus, OH 43210 USA

## Abstract

In nature, pathogens live and interact with other microorganisms on plant tissues. Yet, the research area exploring interactions between bacteria-fungi and microbiota-plants, within the context of a pathobiome, is still scarce. In this study, the impact of olive knot (OK) disease caused by the bacteria *Pseudomonas savastanoi* pv. *savastanoi* (Psv) on the epiphytic and endophytic fungal communities of olive tree twigs from three different cultivars, was investigated in field conditions. The ITS-DNA sequencing of cultivable fungi, showed that OK disease disturbs the resident fungal communities, which may reflect changes in the habitat caused by Psv. In particular, a reduction on epiphyte abundance and diversity, and changes on their composition were observed. Compared to epiphytes, endophytes were less sensitive to OK, but their abundance, in particular of potential pathogens, was increased in plants with OK disease. Host genotype, at cultivar level, contributed to plant fungal assembly particularly upon disease establishment. Therefore, besides fungi - Psv interactions, the combination of cultivar - Psv also appeared to be critical for the composition of fungal communities in olive knots. Specific fungal OTUs were associated to the presence and absence of disease, and their role in the promotion or suppression of OK disease should be studied in the future.

## Introduction

The above-ground plant parts (phyllosphere) are naturally inhabited by a great diversity of microbes^1^. Plant health can be negatively impacted by the presence of plant pathogens in these microbial communities, but some microbes may also provide direct or indirect pathogen protection^2^. Indeed, many plant pathogens do not act alone^3^. Their disease potential is mediated by different microbe interactions occurring within the pathobiome, including microbial cooperation or antagonistic interactions^4–6^. Such findings have been mostly recognized from interaction studies involving microorganisms from the same kingdom, either bacteria or fungi^2^. The importance of bacterial-fungal interactions for host health has been widely demonstrated for humans^7,8^, but their relevance for plant health is still scarce. Few examples indicate that bacterial-fungal interactions can result in enhanced or decreased pathogenicity of one partner towards their plant host^6,9^. Thus, both microbial kingdoms are expected to work in association, through competition or cooperation, for a more effective interaction with plant host^10^.

Most studies about microbial interactions in the phyllosphere have only considered a single plant genotype^9^, even though plant genotype has been increasingly recognized to be a key determinant of phyllosphere microbiota composition^11^. This raises the question whether host plant genotype can affect the outcome of microbial interactions occurring between the incoming pathogens and their resident microbiota. Also, previous studies were mainly performed using artificial and simplified bioassays, which only partially reflect the complex phyllosphere conditions, and were exclusively focused on interactions among either endophytic or epiphytic communities^9^.

In the present study, we used the olive knot disease caused by the bacterial pathogen *Pseudomonas savastanoi* pv. *savastanoi* (Psv) as a model system for understanding interactions between pathogenic bacteria and epi- or endophytic fungal communities, taking place on host plants (olive trees) in the field. Olive knot (OK) is one of the most important worldwide olive tree (*Olea europaea* L.) diseases, causing serious losses in terms of production and olive oil quality^12,13^. This disease is characterized by the formation of overgrowth tissues (knots) in the aerial part of olive trees, mainly on twigs and branches^13^. We chose these knots caused by Psv as a model for studying bacterial-fungal interactions because they have already been shown to provide a special niche for studying microbial multispecies interactions^14^. Indeed, recent studies indicated that some non-pathogenic bacteria, namely *Erwinia toletana*, *Pantoea agglomerans* and *Erwinia oleae*, are frequently associated with Psv in olive knots and effectively cooperate with the pathogen for increasing disease severity^15,16^. Compared with bacterial communities, the fungal community composition in olive knots remains unknown, as well as the way the bacterial pathogen interacts and impacts this fungal community. Using this model system, the simultaneous study of interactions occurring within members of epiphytic or endophytic microbial communities is possible, due to the recognized ability of Psv to live as an epiphyte or endophyte in the olive phyllosphere^12^. Interactions occurring within epiphytic members are of particular interest since the infection of olive tree is believed to be cause by the epiphytic Psv^17^. The availability of olive cultivars with different susceptibility levels to olive knot^12^, which could simultaneously present asymptomatic twigs and knots in the same olive tree, also makes this pathosystem a good model for studying microbial interactions.

With this work, we specifically want to answer the following questions: i) What is the effect of olive knot disease, tree genotype (at cultivar level), and their interaction on fungal communities of twigs? ii) Are these effects identical on epi- and endophytic fungal communities? iii) Is there any fungal consortia associated with olive knot disease and/or host susceptibility? To accomplish this, the composition of both epiphytic and endophytic fungal communities, associated to symptomless twigs and knots from three distinct olive cultivars, were investigated under field conditions. Fungal communities were assessed through PCR identification of culturable isolates. These isolates will be very useful to study the mechanisms of interactions among the most prominent fungi, the pathogen Psv and host plant, and their implication in the control of OK disease. This work is the first step for ascertaining the role of such fungi on OK disease establishment/development in olive tree.

## Results

In this study, 179 fungal OTUs belonging to two phyla, 47 families, and 89 genera were identified as inhabitants of olive tree twigs. Ascomycota was the most abundant phylum, accounting for 97.2% of the isolates (Supplementary Fig. S1). The remaining isolates belonged to Basidiomycota. *Fusarium* (Nectriaceae), *Alternaria* (Pleosporaceae), and *Cladosporium* (Cladosporiaceae) were the most abundant genera, accounting together with 43% of total isolates (Supplementary Fig. S2; Table S1). Indeed, *Alternaria* was the most frequently isolated in the endophytic community, whereas *Cladosporium* was dominant in the epiphytic community (Supplementary Fig. S2; Table S1). Epiphytes were significantly more abundant (*P* < 0.002) and diverse (*P* < 0.05) than endophytes.

### Olive knot disease affects mostly the epiphytic fungal diversity

**Figure 1:**
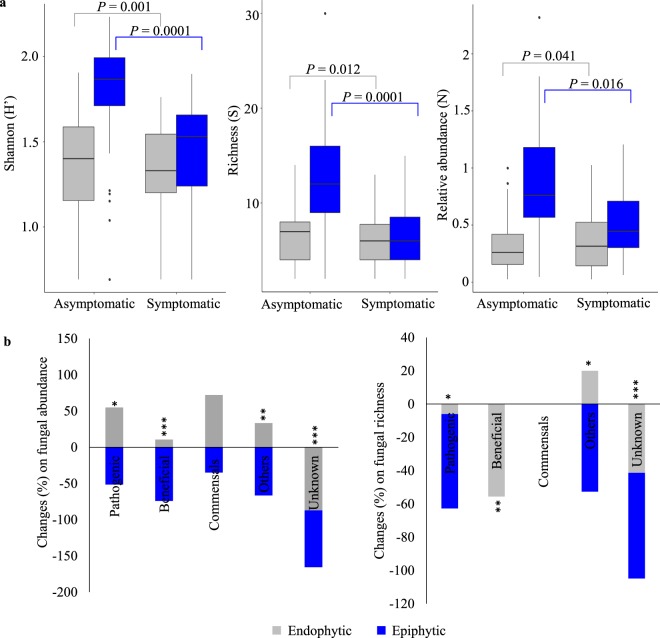
Comparison of fungal diversity between asymptomatic and OK-symptomatic twigs, either within endophytic or epiphytic communities. (**a**) Diversity at community level evaluated by determining abundance, richness and by using Shannon–Wiener index. Box plots depict medians (central horizontal lines), the inter-quartile ranges (boxes), 95% confidence intervals (whiskers), and outliers (black dots). Significant differences between pairs of values are represented over horizontal lines. (**b**) Changes (%) on fungal abundance and richness for each functional group, occurring on OK-symptomatic twigs in relation to asymptomatic twigs. Asterisks indicate significant differences between these two samples (*P < 0.05, **P < 0.01, ***P < 0.001).

The diversity of epiphytic and endophytic fungal communities varied significantly between asymptomatic (stems) and OK-symptomatic (knots) twigs, but differences were greater for epiphytes (Fig. 1a). Epiphytic fungi showed a greater decline in abundance (up to 1.7-fold), richness (up to 1.9-fold) and diversity (up to 1.3-fold) in symptomatic twigs (in relation to asymptomatic twigs) than endophytic fungi. In particular, a reduction in abundance of epiphytes belonging to Pleosporaceae, Chaetomiaceae, and Aspergillaceae was evident in symptomatic twigs, and in a lesser extent to Pyronemataceae, Phaeomoniellaceae, Hypocreaceae and Valsariaceae families (Supplementary Fig. S3). In addition, thirteen epiphytic families disappeared in symptomatic twigs. There was a greater abundance (up to 1.2-fold) of fungal endophytes in OK-symptomatic versus asymptomatic twigs, but a smaller reduction in richness and diversity (up to 1.1-fold) (Fig. 1a). The increase in abundance was mainly due to the Nectriaceae family (Supplementary Fig. S3), particularly the *Fusarium* genus (Supplementary Fig. S2; Table S1). Additionally, seven endophytic families, found in asymptomatic twigs, disappeared in symptomatic twigs (Supplementary Fig. S3).

When we examined fungal guilds, we found the abundance of most epiphytic trophic groups decreased significantly on symptomatic twigs, while the abundance of most endophytic fungi increased (Fig. 1b). Changes in the richness of fungal trophic groups promoted by OK disease were greater for the epiphytic community, where a great decrease in the pathogenic group was observed. Within endophytes, a significant decrease in the richness of beneficial fungi was observed in symptomatic twigs.

### Olive knot disease affects mostly the fungal diversity of the most OK-tolerant cultivar

**Figure 2:**
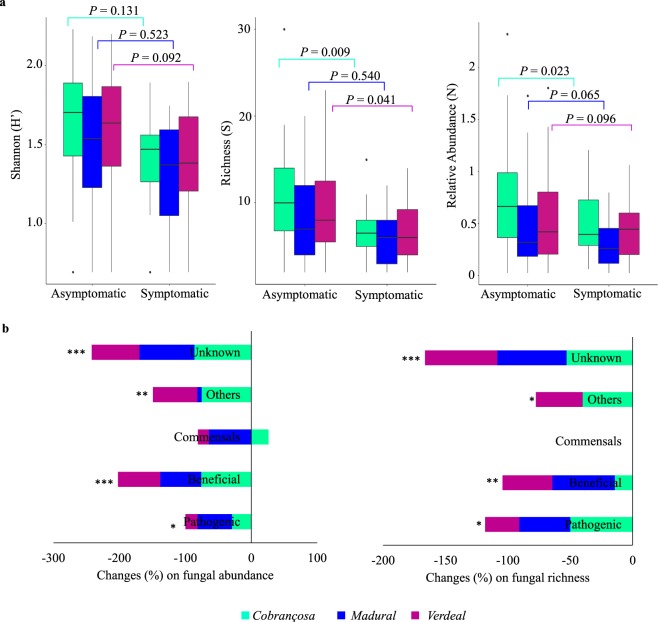
Comparison of fungal diversity between asymptomatic and OK-symptomatic twigs within each olive tree cultivar (*Cobrançosa*, *Madural* and *Verdeal Transmontana*). (**a**) Diversity at community level by determining abundance, richness and by using Shannon–Wiener index. Box plots depict medians (central horizontal lines), the inter-quartile ranges (boxes), 95% confidence intervals (whiskers), and outliers (black dots). Significant differences between pairs of values are represented over horizontal lines. (**b**) Changes (%) on fungal abundance and richness for each functional group, occurring on OK-symptomatic twigs in relation to asymptomatic twigs. Asterisks indicate statistically significant differences between these two samples (*P < 0.05, **P < 0.01, ***P < 0.001).

Fungal communities inhabiting asymptomatic twigs varied by cultivar and disease. We found the highest abundance and fungal diversity (*P* < 0.05) in the most OK tolerant cultivar (cv. *Cobrançosa*) followed by cvs. *Madural* and *Verdeal Transmontana* (Fig. 2a). Fungal communities of each cultivar were differentially affected by OK disease. Knots of cv. *Cobrançosa* lead to a significantly greater loss of both fungal abundance and richness (up to 1.6-fold, in relation to asymptomatic twigs) than knots in cvs. *Madural* (up to 1.4-fold) or *Verdeal Transmontana* (up to 1.1-fold). Most of the lost isolates in knots belonged to the Pezizaceae (for cv. *Cobrançosa*), Chaetomiaceae (for cv. *Madural*) and Gnomoniaceae (for cv. *Verdeal Transmontana*) (Supplementary Fig. S3). We observed an increase in abundance of Nectriaceae and Pestalotiopsidaceae families in the symptomatic twigs across all cultivars, and an increase in the Mycosphaerellaceae for the most OK-susceptible cultivar (cv. *Verdeal Transmontana*).

When fungal OTUs were divided into functional categories, host genotype differences were also detected (Fig. 2b). For all cultivars, a significant decline in abundance and richness of all functional groups was observed in symptomatic twigs in relation to asymptomatic twigs, with the exception of commensals. Abundance of beneficial fungi and richness of pathogens underwent greater declines on cv. *Cobrançosa*, while pathogenic and commensal fungal abundance, as well as richness of beneficial fungi, were greatly reduced in cv. *Madural*.

### Disease was the major driver of the fungal community composition, whereas host cultivar shaped these communities in symptomatic twigs

**Figure 3:**
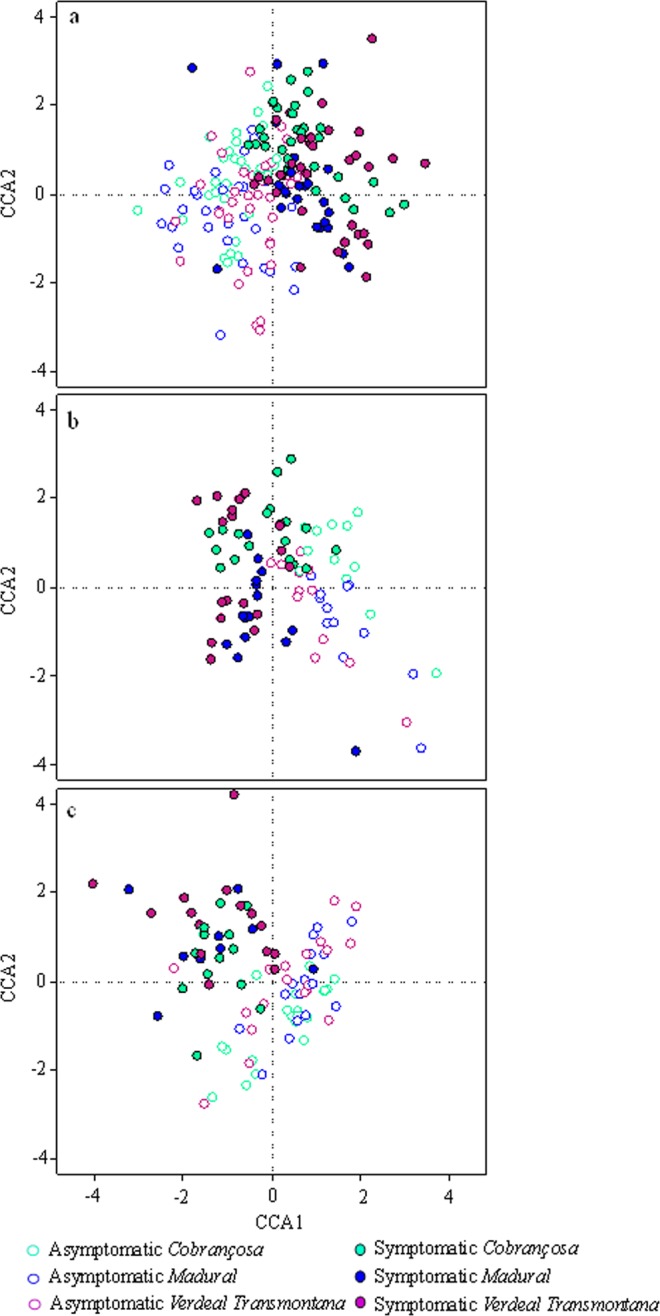
Canonical correspondence analysis (CCA) ordination showing the dispersion of individual OTUs within total (**a**), endophytic (**b**) and epiphytic (**c**) fungal communities relative to the presence/absence of OK-twig symptoms and olive tree cultivar (*Cobrançosa*, *Madural* and *Verdeal Transmontana*).

As revealed by the fungal clustering in the CCA analysis, the fungal composition in twigs was mainly driven by OK disease rather than by the host genotype (Fig. 3). Results from ANOVA (Table S2) and ANOSIM (Supplementary Table S3) confirm that the presence/absence of OK symptoms had the greatest influence on fungal species composition (*P* = 0.005). Disease explained 3.9%, 5.6% and 5.9% of total, endophytic and epiphytic fungal species variation, respectively; whereas host genotype only explained 0.5%, 1.1% and 1.3% of total, endophytic and epiphytic fungal species variation, respectively (Supplementary Table S2). The effect of host genotype on species composition was greater in symptomatic (*P* = 0.005) than in asymptomatic (*P* = 0.049) twigs, explaining 2.8% and 1.5% of species composition variance, respectively (Supplementary Table S2). Differences on fungal species composition between asymptomatic and symptomatic twigs (Supplementary Table S3) were especially noticed in fungal communities of cv. *Cobrançosa*, in particular for epiphytic community (R = 0.498, *P* = 0.001). The interaction between OK disease and host genotype was also shown to influence significantly the overall composition of the fungal community (*P* = 0.010) and the composition of endophytic fungal community (*P* = 0.005). In contrast, epiphyte composition was not impacted by the interaction between OK disease and host genotype (data not shown).

### Specific fungal signatures were detected for the asymptomatic vs. symptomatic twigs

**Figure 4:**
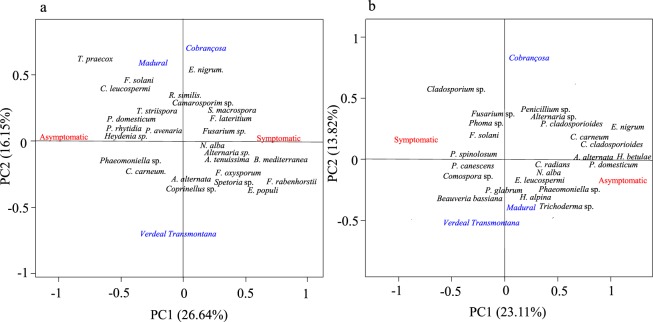
Principal component analysis (PCA) of endophytic (**a**) and epiphytic (**b**) fungal communities, inhabiting asymptomatic and OK-symptomatic twigs from different olive cultivars (*Cobrançosa*, *Madural* and *Verdeal Transmontana*). This analysis was performed with preselected fungal OTUs by the random forest analysis.

Our previous analyses indicate that OK disease plays an important role on fungal community assemblages, suggesting the existence of a fungal consortium associated to either asymptomatic or OK-symptomatic twigs. To test this hypothesis, a random forest analysis was performed to provide a ranking of the relative importance of each endophyte and epiphyte OTU for distinguishing asymptomatic from OK-symptomatic twigs (Supplementary Fig. S4). The most distinguishing fungal OTUs were selected and then used to perform a PCA, in order to identify which could be potentially related to OK disease and cultivar (Fig. 4). Among the selected OTUs, some were specifically associated with a particular set of twigs, either asymptomatic or symptomatic. *Heydenia* sp., *Phaeomoniella* sp., *Plectania rhytidia*, *Pyronema domesticum*, and *Chromelosporium carneum*, in the endophytic community, as well as *Alternaria alternata*, *Pyronema domesticum*, *Hyalodendriella betulae*, *Epicoccum nigrum*, *Cladosporium cladosporioides*, *Coprinellus radians*, *Neofabraea alba*, *Trichoderma* sp. in the epiphytic community, often occur together and are highly associated with asymptomatic twigs. On the other hand, *Fusarium* sp., *Fusarium lateritium*, *Biscogniauxia mediterranea*, *Fusarium oxysporum*, *Neofabraea alba*, *Alternaria* sp., and *Alternaria tenuissima*, in the endophytic community, as well as *Penicillium spinolosum*, *Penicillium canescens* and *Comospora* sp., in the epiphytic community, were simultaneously found on symptomatic twigs (knots). However, a clear association of these fungal OTUs and olive cultivars was not found. In order to discover significant associations between fungal OTUs of asymptomatic/symptomatic twigs and olive cultivars, a species indicator analysis was carried out using preselected OTUs by the random forest analysis. The best indicator OTUs (*IndVal* > 0.70) of asymptomatic and symptomatic twigs were found in cv. *Cobrançosa* (Table 1). Curiously, *H. betulae* was identified as an epiphyte indicator of asymptomatic twigs for all three cultivars, while the endophyte *Fusarium* was found to be a good indicator of symptomatic twigs for all three cultivars.

**Table 1:** Endophytic and epiphytic fungal indicator OTUs in asymptomatic and OK-symptomatic twigs from olive tree cultivars *Cobrançosa*, *Madural* and *Verdeal Transmontana*.

			Indicator species	A	B	*IndVal*
*Endophytic*	*Cobrançosa*	Asymptomatic	*Chromelosporium carneum*	0.97	0.72	0.84
		Symptomatic	*Fusarium lateritium*	0.98	0.91	0.95
			*Fusarium* sp.	0.81	0.66	0.73
	*Madural*	Aymptomatic	*Pyronema dosmesticum*	1.00	0.52	0.72
		Symptomatic	*Alternaria alternata*	0.90	0.60	0.74
			*Fusarium lateritium*	1.00	0.30	0.55
	*Verdeal*	Asymptomatic	*Chromelosporium carneum*	1.00	0.55	0.74
			*Pyronema dosmesticum*	1.00	0.27	0.52
		Symptomatic	*Fusarium oxysporum*	1.00	0.44	0.66
*Epiphytic*	*Cobrançosa*	Asymptomatic	*Cladosporium cladosporioides*	0.87	0.85	0.87
			*Alternaria* sp.	0.72	0.81	0.77
			*Hyalodendriella betulae*	0.88	0.57	0.71
			*Biscogniauxia mediterranea*	0.81	0.52	0.65
			*Alternaria alternata*	1.00	0.38	0.61
			*Pyronema dosmesticum*	1.00	0.38	0.61
		Symptomatic	*Cladosporium* sp.	0.76	0.78	0.77
	*Madural*	Asymptomatic	*Coniozyma leucospermi*	1.00	0.43	0.66
			*Hyalodendriella betulae*	1.00	0.43	0.66
		Symptomatic	*Phoma* sp.	0.91	0.33	0.55
	*Verdeal*	Asymptomatic	*Penicillium* sp.	0.95	0.50	0.69
			*Hyalodendriella betulae*	0.86	0.55	0.69
			*Phoma aloes*	1.00	0.35	0.59
			*Heydenia alpina*	1.00	0.30	0.54
			*Phaeomoniella* sp.	1.00	0.30	0.54
		Symptomatic	*Beauveria bassiana*	0.83	0.37	0.56
			*Comospora* sp.	1.00	0.33	0.56

A - Specificity (*i.e*. uniqueness to a particular habitat), B – Sensitivity (*i.e*. frequency within that particular habitat).

## Discussion

A great diversity of fungal epiphytes and endophytes (in total 179 OTUs) was found in twigs/knots of olive tree. This fungal community was studied based on cultivation-dependent method and therefore it is expected that twigs/knots harbor a much larger diversity. Indeed, such approach has limitations due to some non-sporulating and non-culturable fungi^18^. Despite the limited coverage of culture-based methods, previous studies have indicated that, the dominant fungal taxa identified by cultivation-independent methods were also detected by culture-dependent methods^19^. In this work, we had used culture-based method because it offers the possibility to isolate and study the fungal strains for their biological features in terms of biological control of olive knot disease and mechanisms involved.

Our comparative community analysis of fungi between asymptomatic and OK-symptomatic twigs showed that OK disease primarily affects the epiphytic fungal community, by decreasing abundance, richness, and diversity, suggesting that Psv may prevent fungal colonization and proliferation on the knot surface. Antifungal activity displayed by other *Pseudomonas* species has been already reported, and applied in the biocontrol of a wide range of fungal phytopathogens^17^. *Pseudomonas* have been shown to produce antibiotics and fungal cell wall degrading enzymes, as well as a competitor for space and nutrient sources^20^. However, very few studies have explicitly examined the antifungal activity promoted by phytopathogenic *Pseudomonas*^9^. There is evidence suggesting phytopathogenic *Pseudomonas* may inhibit the growth and extract nutrients from filamentous fungi in the phyllosphere^21^. Thus, Psv may also suppress fungal colonization/proliferation on olive knot surface in a similar manner. Compared to epiphytes, the endophytic fungal diversity and richness in knots was less impacted, suggesting endophytes are less sensitivity to OK disease. But the fungal endophyte abundance increased in olive knots, suggesting an unexpected stimulatory effect of Psv on endophytic fungi, particularly *Pseudocercospora* spp. (Mycosphaerellaceae family), which are well-recognized plant pathogens on a wide range of plant hosts, including olive trees^22^. This increasing abundance in the presence of Psv could result from the production of specific compounds by the pathogenic bacteria that could benefit endophytic fungi^9^. Effects of *Pseudomonas* on fungal growth and density stimulation have already been demonstrated in a number of settings, including mushroom formation^23,24^ and human infections^25^.

The effect of OK disease on fungal richness and abundance was greater in the OK-tolerant cv. *Cobrançosa*, compared to both OK-susceptible cultivars. Probably there is a cultivar interaction that promoted the Psv antifungal activity involving cv. *Cobrançosa*, either due to the cultivar chemical composition or because this cultivar had a different initial fungal community. This effect has been previously observed in medicine^26^. Clinical research on the role of *Candida*–bacteria interactions in disease indicated that host’s microbial community might play an important role in the preparation of the fungus for its role in the infection^26^. In our study, a strong decrease in *Chromelosporium carneum* (Pezizaceae) abundance was observed, suggesting that this species is highly sensitive to OK disease aspects in the plant. Future work is needed to validate *C. carneum* inhibition by Psv and to elucidate the importance of *C. carneum* abundance in the knot habitat.

The decline in both fungal richness and abundance between asymptomatic and symptomatic twigs was primarily driven by less studied fungal OTUs, including the genera *Heydenia, Hyalodendriella*, *Masonia*, *Ochrocladosporium* or *Prosthemium* (data not shown). These results open the field for exploring an untapped diversity with potential to benefit olive tree health.

Beneficial and pathogenic fungi were affected by OK disease, but in different ways depending on the type of fungal community (endophytic or epiphytic). On the knot surface, pathogenic fungi decreased in abundance in almost the same proportion as beneficial fungi, indicating that OK disease restricted the growth of both functional groups to the same degree. In contrast, in the knot interior, an increase in pathogenic fungal abundance was accompanied by a decrease in beneficial fungal richness. Some of the lost beneficial fungi were members of *Epicoccum*, *Cladosporium*, and *Penicillium* genera that included antagonists and disease-protective fungi^27–29^. The decline of these fungal genera in olive knots suggests they can potentially limit or prevent OK disease. Pathogens with increased abundance included mostly non-pathogens of olive tree. The potentially role of these beneficial and pathogenic groups in the development of OK disease must be studied in future works.

The composition of the fungal community of olive twigs was primarily impacted by OK disease. This may be due to the Psv ability to shape and change the shared environment either to the benefit or to detriment of certain fungal species^30^. Indeed, Psv has the ability to form biofilm^31^, which represent a hotspot for microbial interactions that locally shape microbial assemblages^2,9^. Bacterial-Psv interactions have already been reported to occur in olive knots, where the presence of Psv is suggested to be essential for the creation and maintenance of a core group of bacterial genera observed in olive knots^16^. These multispecies interactions observed among bacteria may also occur between Psv bacteria and fungi. In addition, pathogen is likely to trigger host susceptibility, which could have an impact on fungal community composition of olive twigs. Indeed, there are some reports indicating that some pathogenic infections can be detrimental to the defense systems predisposing the plant to subsequent secondary infections^32^. For instances, infection by *Pseudomonas syringae* was showed to render *Arabidopsis* plants more vulnerable to invasion by the necrotrophic *Alternaria brassicicola*^33^.

The cultivar affects mostly the fungal community composition of symptomatic twigs (*i.e*. knots) compared to the ones of asymptomatic twigs. Thus, the interaction of cultivar and Psv seems to influence the establishment of fungal communities in olive knots, likely because host plants play a role in recruiting fungi upon OK disease establishment, as previously showed in the *Arabidopsis thaliana* rhizosphere upon foliar pathogen attack^34^. The composition of endophytic fungal community in olive twigs was affected by the interaction between OK disease and cultivar, thus reinforcing the hypothesis of recruitment of fungi by the plant upon Psv attack.

Our data revealed that a consortium of fungal OTUs is associated with asymptomatic or symptomatic twigs of each cultivar. The most parsimonious assumption is that these consortia probably have relevance to olive tree health. The best indicator OTUs of asymptomatic twigs are antagonists toward plant pathogens (*C. cladosporioides* in cv. *Cobrançosa*^35^), and fungi with unknown biological function (*e.g*. *P. domesticum* in cv. *Madural*, *C. carneum* in cv. *Verdeal Transmontana*, and *H. betulae* in the three cultivars). *Alternaria* sp. was also found to be indicative of asymptomatic twigs in cv. *Cobrançosa*. The genus *Alternaria* includes both plant-pathogenic and saprophytic species, and is one of the most well-known fungal genera producers of diverse secondary metabolites, including toxins^36^ and antimicrobial compounds^37^.

The best indicator OTUs for olive knots comprise members of *Fusarium*, a genus including common plant pathogens^38,39^. This taxonomic group has been described as “pathogen facilitators” that may aid pathogen infection of plant hosts or increase disease severity^40^. A strong association was also detected between *A. alternata* and knots of cv. Madural. This is a generalist saprobe fungal species, but also contains several variants, which cause necrotic diseases on different plants^41^. *Fusarium* and *Alternaria* genera include fungal species that have been recently reported to cause olive tree diseases with minor importance^42,43^. The role of these fungal taxa associated to either asymptomatic or symptomatic twigs, on olive tree’s defense against OK disease remains a topic for further study.

In summary, our study indicates that OK disease caused by the bacterium Psv alters the resident fungal community of olive twigs in terms of species composition, abundance and richness. This effect was strongest for epiphytes. In the interior of olive knots, Psv seems to shape fungal assemblages possibly by promoting pathogens. Host plant genotype is also likely to structure olive knot-associated fungal communities. Specific fungal signatures were detected for asymptomatic and symptomatic twigs, suggesting an important role of the fungal community in OK disease establishment and development. These results represent an important step forward in understanding the complexity of interactions between bacteria-fungi, and host-microbe interactions, which is needed for predicting and suppressing OK disease.

## Methods

### Plant sampling

Plant collection was performed during spring 2014, in three olive orchards located in Mirandela, Northeast of Portugal, at coordinates N 41° 32.593′; W 07° 07.445′ (orchard 1), N 41° 32.756′; W 07° 07.590′ (orchard 2) and N 41° 29.490′; W 07° 15.413′ (orchard 3). Each orchard comprised olive trees from three cultivars, planted at 7 × 7 m spacing, with different levels of susceptibility to olive knot disease: cv. *Cobrançosa* is moderately tolerant, cv. *Madural* is moderately susceptible and cv. *Verdeal Transmontana* is the most susceptible. Their susceptibility was confirmed by estimating OK disease incidence simultaneously to sample collection. The levels of disease incidence (%), determined by the percentage of infected twigs, were indeed lower in cv. *Cobrançosa* (7.3 ± 3.2) when compared to cvs. *Madural* (13.4 ± 6.7) and *Verdeal Transmontana* (17.2 ± 9.8). Twigs were sampled from randomly selected seven olive trees of each cultivar, from which asymptomatic and OK-symptomatic (with 3–4 knots) twigs were collected from two cardinal orientations (north and south), at 1.5–2 m above ground height.

### Fungal isolation

From each tree, stem and knot segments (N = 5 each, with around 1 gram of weight/each) were randomly selected from asymptomatic and symptomatic twigs, respectively. These plant tissues were used for isolation of fungal epiphytes and endophytes. Fungal epiphytes were isolated by the plate dilution method, onto Potato Dextrose Agar (PDA, Difco) and Plate Count Agar (PCA, Himedia) media, supplemented with 0.01% (w/v) chloramphenicol (Oxoid), following the procedure described by Gomes *et al*.^44^. The number of epiphytes was expressed as log CFU/cm^2^, *i.e*. the number of individual colonies of fungi adhered to stem/knot surface. To estimate plant tissues surface, a cylinder equation [A = 2πr^2^ + h (2πr)] was used, in which A is the area, r and h are the radius and height of the stem/knot segments, respectively. The average stem/knot segments area of cvs. *Cobrançosa*, *Madural* and *Verdeal Transmontana* were 11.2 ± 0.9, 10.8 ± 1.7 and 11.9 ± 1.8 cm^2^, respectively.

Endophytic fungi were isolated from the same stem/knot segments used to isolated epiphytes, following the procedure described by Martins *et al*.^45^. Briefly, after surface disinfection, each stem/knot was cut in segments (*ca*. 4–5 mm), which were transferred to the same culture media used for epiphyte isolation. Validation of the surface sterilization procedure was done by imprinting the surface of sterilized twigs onto PDA and PCA media. A total of 7,440 plant tissue segments were used to isolated fungal endophytes. Fungal colonies were subcultured on fresh medium until pure epi/endophytic cultures were obtained.

### Fungal identification

We first separated fungal isolates based on their morphological features (both colony shape and microscopic morphology). Then we selected three representative isolates of each morphotype for molecular identification by sequencing the internal transcribed spacer (ITS) region of nuclear ribosomal DNA (rDNA). Total genomic DNA was extracted from harvested mycelium or spores using the *REDExtract-N-Amp*^*™*^ *Plant PCR kit* (Sigma, Poole, UK). The ITS region (ITS1, 5.8S, ITS2) was amplified using ITS1/ITS4 or ITS5/ITS4 primers sets^46^ in a PCR protocol previously described by Oliveira *et al*.^47^. The amplified products (~650 bp) were purified and sequenced using Macrogen Inc. (Seoul, South Korea) services. The obtained DNA sequences were analyzed with *DNASTAR v.2.58* software, and fungal identification was performed using the NCBI database (http://www.ncbi.nlm.nih.gov) and BLAST algorithm^44^. The obtained sequences are available at GenBank with the following accession numbers: KU324941-KU325040; KU325041-KU325240; KU325241-KU325457. Each operational taxonomic unit (OTU) was taxonomically classified according to the *Index Fungorum Database* (www.indexfungorum.org). Pure cultures of each identified isolate were preserved and deposited in the culture collection of the Polytechnic Institute of Bragança (School of Agriculture).

### Effect of OLS disease and host genotype on fungal diversity

The effect of OK disease or host genotype in twig fungal diversity was analyzed at both community and functional group levels. We evaluated *community level* diversity by determining the abundance (number of isolates), richness (number of different OTUs), and computing the Shannon–Wiener (H’) index in *Species Diversity and Richness* v. 4.0^48^. Results are presented as the mean of replicates (N = 21, for each cultivar). We evaluated *functional group* by placing identified fungi into functional categories (commensal, beneficial and pathogenic) according to the description of fungal endophytes by Hardoim *et al*.^49^. The commensal group is comprised of fungi that do not have any apparent effect on host plants, whereas beneficial fungi can protect host plants against pathogens and pests and/or promote plant growth. The pathogen group includes latent plant pathogens. Fungal OTUs belonging to Incertae sedis or other functional groups, were categorized as “unknown fungi” or “other”, respectively. The several taxa were placed in functional categories using expert knowledge (Table S4). After grouping, the relative abundance and richness of each functional group across asymptomatic and OK-symptomatic twigs was determined. To determine differences in fungal community or functional diversity among twigs, a one-way analysis of variance (ANOVA) with *SPSS* v.20 was performed, with multiple comparisons according to Tukey (*P* < 0.05).

### Data analysis

A combination of univariate and multivariate methods were used to identify potential fungal OTUs that differentiate olive tree cultivars (cvs. *Cobrançosa*, *Madural* and *Verdeal-Transmontana*) and twig status (asymptomatic and symptomatic). All statistical analyses were performed in *R*^50^.

#### Effect of OK disease and host genotype on fungal community structure

A canonical correlation analysis (CCA) was used to find possible correlations among surveyed cultivars or twig status with the identified fungal communities (endophytes, epiphytes and total). All data were log_2_(x + 1) transformed for standardization. The CCA was performed using the “CCorA” function in the *vegan* package^51^. One-way analysis of variance (ANOVA) was performed with the “anova” function to determine statistical significant differences among cultivars and twig status, on endophytic, epiphytic and total fungal communities. Variation partitioning was used to calculate community dissimilarity (%), according to different explanatory variables (olive cultivar and presence/absence of OK-symptoms). This analysis was also performed with the *vegan* package using the “varpart” function^52^. A one-way analysis of similarity (ANOSIM) was used to test statistic differences between fungal groups separated by the CCA, using the Bray–Curtis distance matrices. This analysis was performed using the “anosim” function in the *vegan* package^51^.

#### Identification of fungal OTUs associated with OK disease and/or host genotype

A random forest analysis using artificial intelligence algorithms was performed to identify the ranking importance of fungal OTUs for distinguishing asymptomatic from OK-symptomatic twigs^53,54^. For each tree grown on a bootstrap sample, the error rate for observations left out of the bootstrap sample was monitored. The *mean decrease in Gini* coefficient indicates the variable contribution for distinguishing between asymptomatic and OK-symptomatic twigs. The ranking species importance explained 81.4% and 84.2% for endophytes and epiphytes, respectively. We then conducted a principal component analysis (PCA) and indicator fungal species analysis using the species pre-selected by the random forest analysis. Both analyses were used to explore the potential associations between fungal OTUs and cultivar/twig status. The PCA was performed by using the *psych* package^55^. The indicator fungal species analysis was conducted using the function “*multipatt*” from *indicspecies* package^56^. We used the Indicator Value Index (*IndVal*) (*IndVal* > 0.5 represents the most constant and specific species) which is defined as the product of two components: “A”, the *specificity* of the species as indicator of the site group and “B”; the *sensitivity* of the species as indicator of the site group^56^.

## Supplementary information

Supplementary Information

## References

[1] Vorholt JA (2012). Microbial life in the phyllosphere. Nat Rev Microbiol.

[2] Hassani MA, Durán P, Hacquard S (2018). Microbial interactions within the plant holobiont. Microbiome.

[3] Rovenich H, Boshoven JC, Thomma BPHJ (2014). Filamentous pathogen effector functions: of pathogens, hosts and microbiomes. Curr. Opin. Plant Biol..

[4] Kemen E (2014). Microbe–microbe interactions determine oomycete and fungal host colonization. Curr. Opin. Plant Biol..

[5] Vayssier-Taussat M (2014). Shifting the paradigm from pathogens to pathobiome: new concepts in the light of meta-omics. Front. Cell Infect. Microbiol..

[6] Jakuschkin B (2016). Deciphering the Pathobiome: Intra- and interkingdom interactions involving the pathogen *Erysiphe alphitoides*. Microb. Ecol..

[7] Morales DK, Hogan DA (2010). *Candida albicans* interactions with bacteria in the context of human health and disease. PLoS Pathog..

[8] Arvanitis M, Mylonakis E (2015). Fungal–bacterial interactions and their relevance in health. Cel Microbiol..

[9] Frey-Klett P (2011). Bacterial-fungal interactions: hyphens between agricultural, clinical, environmental, and food microbiologists. Microbiol. Mol. Biol. Rev..

[10] Hacquard S (2017). Commentary: Microbial Small Talk: Volatiles in Fungal–Bacterial. Front. Microbiol..

[11] Bodenhausen N, Bortfeld-Miller M, Ackermann M, Vorholt JA (2014). A Synthetic community approach reveals plant genotypes affecting the phyllosphere microbiota. PLoS Genet..

[12] Quesada, J.M., Penyalver, R. & López, M.M. Epidemiology and control of plant diseases caused by phytopathogenic bacteria: The case of Olive knot disease caused by *Pseudomonas savastanoi* pv. *savastanoi in**Plant Pathology* (ed. Cumagun, J.C.) 209–326 (InTech, 2012).

[13] Ramos C, Matas M, Bardaji L, Aragón IM, Murillo J (2012). *Pseudomonas savastanoi* pv. *savastanoi*: some like it knot. Mol. Plant Pathol..

[14] Buonaurio R (2015). The olive knot disease as a model to study the role of interspecies bacterial communities in plant disease. Front. Plant Sci..

[15] Hosni T (2011). Sharing of quorum-sensing signals and role of interspecies communities in a bacterial plant disease. ISME J..

[16] Passos da Silva D (2014). Bacterial multispecies studies and microbiome analysis of a plant disease. Microbiol.

[17] Quesada JM (2010). Dissemination of *Pseudomonas savastanoi* pv. *savastanoi* populations and subsequent appearance of olive knot disease. Plant Pathol.

[18] Sun X, Guo L-D (2012). Endophytic fungal diversity: review of traditional and molecular techniques. Mycology.

[19] Liu T, Greenslade A, Yang S (2017). Levels of rhizome endophytic fungi fluctuate in *Paris polyphylla* var. *yunnanensis* as plants age. Plant Diver.

[20] Someya, N., Ikeda, S. & Tsuchiya, T. *Pseudomonas* inoculants as agents for plant disease management in *Bacteria in Agrobiology: Disease Management* (Maheshwari, D.K. ed.) 495 (Springer-Verlag, 2013).

[21] Wichmann G, Sun J, Dementhon K, Glass NL, Lindow SE (2008). A novel gene, phcA from *Pseudomonas syringae* induces programmed cell death in the filamentous fungus *Neurospora crassa*. Mol. Microbiol..

[22] Crous PW (2013). Phylogenetic lineages in *Pseudocercospora*. Studies Mycol..

[23] Rainey P, Cole A, Fermor T, Wood D (1990). A model system for examining involvement of bacteria in basidiodome initiation of *Agaricus bisporus*. Mycol. Res..

[24] Cho YS, Kim JS, Crowley DE, Cho BG (2003). Growth promotion of the edible fungus *Pleurotus ostreatus* by fluorescent pseudomonas. FEMS Microbiol. Lett..

[25] Briard B, Heddergott C, Latgé J (2016). Volatile compounds emitted by *Pseudomonas aeruginosa* stimulate growth of the fungal pathogen *Aspergillus fumigatus*. mBio.

[26] Wargo MJ, Hogan DA (2006). Fungal-bacterial interactions: a mixed bag of mingling microbes. Curr. Opin. Microbiol..

[27] Dzoyem JP (2017). Cytotoxicity, antioxidant and antibacterial activity of four compounds produced by an endophytic fungus *Epicoccum nigrum* associated with *Entada abyssinica*. Rev. Bras. Farmacogn..

[28] Khan IH (2016). Cytotoxic and antibacterial naphthoquinones from an endophytic fungus, *Cladosporium* sp. Toxicol. Rep..

[29] Jouda JB (2016). Anticancer and antibacterial secondary metabolites from the endophytic fungus *Penicillium* sp. CAM64 against multi-drug resistant Gram-negative bacteria. Afr. Health Sci..

[30] McNally L, Brown S (2015). Building the microbiome in health and disease: niche construction and social conflict in bacteria. Philos. Trans. R. Soc. Lond. B Biol. Sci..

[31] Rodríguez-Moreno L, Jiménez AJ, Ramos C (2009). Endopathogenic lifestyle of *Pseudomonas savastanoi* pv. *savastanoi* in olive knots. Microb. Biotechnol..

[32] Abdullah AS (2017). Host–Multi-Pathogen warfare: pathogen interactions in co-infected plants. Front Plant Sci..

[33] Spoel SH, Johnson JS, Dong X (2007). Regulation of tradeoffs between plant defenses against pathogens with different lifestyles. Proc Natl Acad Sci USA.

[34] Berendsen RL (2018). Disease-induced assemblage of a plant-beneficial bacterial consortium. ISME J..

[35] Köhl J, Scheer C, Holb IJ, Masny S, Molhoek W (2015). Toward an integrated use of biological control by *Cladosporium cladosporioides* H39 in Apple Scab (*Venturia inaequalis*) management. Plant Dis..

[36] Dang HX, Pryor B, Peever T, Lawrence CB (2015). The *Alternaria* genomes database: a comprehensive resource for a fungal genus comprised of saprophytes, plant pathogens, and allergenic species. BMC Genomics.

[37] Vaz AB (2009). Antimicrobial activity of endophytic fungi associated with Orchidaceae in Brazil. Can. J. Microbiol..

[38] Santori A, Vitale S, Luongo L, Belisario A (2010). First report of *Fusarium lateritium* as the agent of Nut Gray Necrosis on hazelnut in Italy. Plant Dis..

[39] Duan C (2016). Identification of pathogenic *Fusarium* spp. causing maize ear rot and potential mycotoxin production in China. Toxins.

[40] Rodriguez-Estrada AE, Jonkers W, Kistler HC, May G (2012). Interactions between *Fusarium verticillioides*, *Ustilago maydis*, and *Zea mays*: An endophyte, a pathogen, and their shared plant host. Fungal Genet. Biol..

[41] Ito K (2004). Dissection of the host range of the fungal plant pathogen *Alternaria alternata* by modification of secondary metabolism. Mol Microbiol.

[42] Trabelsi R (2017). Morphological and molecular characterization of *Fusarium* spp. associated with olive trees dieback in Tunisia. 3 Biotech.

[43] Basim E, Basim H, Abdulai M, Öztürk N (2017). Identification and characterization of *Alternaria alternata* causing leaf spot of olive tree (*Olea europaea*) in Turkey. Crop Prot..

[44] Gomes T, Pereira JA, Benhadi J, Lino-Neto T, Baptista P (2018). Endophytic and epiphytic phyllosphere fungal communities are shaped by different environmental factors in a Mediterranean ecosystem. Microb. Ecol..

[45] Martins F, Pereira JA, Bota P, Bento A, Baptista P (2016). Fungal endophyte communities in above- and belowground olive tree organs and the effect of season and geographic location on their structures. Fungal Ecol..

[46] White, T.J., Bruns, T., Lee, S. & Taylor, J. Amplification and direct sequencing of fungal ribosomal RNA genes for phylogenetics in *PCR Protocols: a guide to methods and applications* (ed. Innis, M.A., Gelfand, D.H., Sninsky, J.J., White, T.J.) 315–322 (Academic Press, 1990).

[47] Oliveira I, Pereira JA, Lino-Neto T, Bento A, Baptista P (2012). Fungal diversity associated to the Olive Moth, *Prays Oleae* Bernard: A Survey for potential entomopathogenic fungi. Microb. Ecol..

[48] Seaby, R. M. & Henderson, P. A. *Species Diversity and Richness Version 4* (Pisces Conservation Ltd., 2006).

[49] Hardoim PR (2015). The hidden world within plants: ecological and evolutionary considerations for defining functioning of microbial endophytes. Microbiol. Mol. Biol. Ver..

[50] R Core Team. *R: A language and environment for statistical computing*. R Foundation for Statistical Computing, http://www.R-project.org/ (2014).

[51] Oksanen, J. *et al*. Vegan: community ecology package in Ordination methods, *diversity analysis and other functions for community and vegetation ecologists* (ed. R package version 2.3–2) (2015).

[52] Legendre, P. & Legendre, L. *Numerical**Ecology* 3rd edition (Elsevier, 2012).

[53] Breiman L (2011). Random forests. Mach. Learn.

[54] Cutler DR (2007). Random Forests for classification in ecology. Ecol. Appl..

[55] Revelle, W. *Psych: procedures for psychological, psychometric, and personality research*. R package 1.6.12. https://cran.r-project.org/web/packages/ psych/ (2017).

[56] Cáceres M, Legendre P, Wiser SK, Brotons L (2012). Using species combinations in indicator value analyses. Methods Ecol. Evol..

