# Supplementary material for: Bacterial disease induced changes in fungal communities of olive tree twigs depend on host genotype
Source: Sci Rep. 2019 Apr 10;9:5882. doi: 10.1038/s41598-019-42391-8 (PMC6458152; doi:10.1038/s41598-019-42391-8)
Supplement: Supplementary file 1 — Supplementary Information [file 41598_2019_42391_MOESM1_ESM.docx]

**Supplementary Information**

**Article title: Bacterial disease induced changes in fungal communities of olive tree twigs depend on host genotype**

**Authors:** Teresa Gomes, José Alberto Pereira, Teresa Lino-Neto, Alison E. Bennett, Paula Baptista

**The following Supplementary Information is available for this article:**

**Fig. S1** - Krona chart of the taxonomic affiliation down to family level of the global fungal community found in olive twigs.

**Fig. S2 -** Frequency (%) of isolates within each genera of fungal epiphytes and endophytes present in asymptomatic and OK-symptomatic twigs from *Cobrançosa*, *Madural* and *Verdeal Transmontana* olive cultivars.

**Fig. S3** - Distribution of families in epiphytic (**a**) and endophytic (**b**) fungal communities present in asymptomatic and OK-symptomatic twigs of *Cobrançosa*, *Madural* and *Verdeal Transmontana* olive cultivars. Square sizes represent the number of fungal isolates (abundance) present in each plant tissue sample.

**Fig. S4** – Ranking of relative importance of each fungal OTUs in distinguishing among asymptomatic and OK-symptomatic twigs within endophytic (**a**) and epiphytic (**b**) fungal communities. *Mean Decrease Gini* value predicts fungal OTUs that distinguish asymptomatic from OK-symptomatic twigs. The highest values represent the best predictors.

**Table S1**- Frequency (%) of isolates within each genera of fungal epiphytes and endophytes present in asymptomatic and OK-symptomatic twigs from Cobrançosa, Madural and Verdeal Transmontana olive cultivars.

**Table S2** - ANOVA analysis to test significant differences (*P*-value) between fungal community groups obtained in the Canonical Correlation Analysis (CCA). Variation partitioning (*Varpart*) was calculated to achieve the total variance explained by each factor. *P*-values in bold are significant.

**Table S3** - Analysis of similarity (ANOSIM), based on Bray-Curtis distance, between fungal communities inhabiting asymptomatic and OK-symptomatic twigs. R-statistics (R) and *P*-values of each variable are included.

**Table S4** – Functional categories of the several fungal operational taxonomic unit (OTU) identified in this work.

**Fig. S1** - Krona chart of the taxonomic affiliation down to family level of the global fungal community found in olive twigs.

**Fig. S2 -** Frequency (%) of isolates within each genera of fungal epiphytes and endophytes present in asymptomatic and OK-symptomatic twigs from *Cobrançosa*, *Madural* and *Verdeal Transmontana* olive cultivars.

**Fig. S3** - Distribution of families in epiphytic (**a**) and endophytic (**b**) fungal communities present in asymptomatic and OK-symptomatic twigs of *Cobrançosa*, *Madural* and *Verdeal Transmontana* olive cultivars. Square sizes represent the number of fungal isolates (abundance) present in each plant tissue sample.

**Fig. S4** – Ranking of relative importance of each fungal OTUs in distinguishing among asymptomatic and OK-symptomatic twigs within endophytic (**a**) and epiphytic (**b**) fungal communities. *Mean Decrease Gini* value predicts fungal OTUs that distinguish asymptomatic from OK-symptomatic twigs. The highest values represent the best predictors.

**Table S1**- Frequency (%) of isolates within each genera of fungal epiphytes and endophytes present in asymptomatic and OK-symptomatic twigs from Cobrançosa, Madural and Verdeal Transmontana olive cultivars.

|  | **Epiphytic** | | | | | | **Endophytic** | | | | | |
| --- | --- | --- | --- | --- | --- | --- | --- | --- | --- | --- | --- | --- |
|  | Asymptomatic | | | Symptomatic | | | Asymptomatic | | | Symptomatic | | |
|  | *Cobrançosa* | *Madural* | *Verdeal* | *Cobrançosa* | *Madural* | *Verdeal* | *Cobrançosa* | *Madural* | *Verdeal* | *Cobrançosa* | *Madural* | *Verdeal* |
| *Alternaria* | 9.8±3.6 | 9.8±3.3 | 9.0±4.2 | 4.9±1.9 | 4.9±1.8 | 2.5±1.1 | 15.3±4.2 | 15.5±4.6 | 24.9±8.4 | 17.8±13.1 | 25.9±14.8 | 28.3±22.7 |
| *Anthostomella* | - | - | - | - | - | - | - | - | 0.6±0.4 | - | - | - |
| *Arthrinium* | 0.6±0.4 | 0.6±0.4 | - | - | - | 2.1±1.3 | - | - | - | - | - | - |
| *Ascobolus* | 0.6±0.3 | 0.6±0.4 | - | 1.3±0.8 | - | - | - | - | - | - | - | - |
| *Aspergillus* | 3.3±0.9 | 3.3±1.2 | 4.3±1.3 | 0.5±0.2 | 1.8±0.8 | 3.7±0.6 | 1.3±0.6 | - | 0.6±04 | 0.5±0.3 | 0.6±0.4 | 0.2±0.1 |
| *Aureobasidium* | - | - | 0.4±0.2 | - | - | - | - | - | - | - | - | - |
| *Beauveria* | 0.7±0.3 | 0.7±0.4 | 0.9±0.5 | 0.9±0.4 | 2.5±1.2 | 4.8±2.6 | - | - | - | - | - | - |
| *Biscogniauxia* | 3.5±1.2 | 3.5±1.7 | 3.3±1.6 | 1.3±0.4 | 4.6±2.2 | 1.7±0.8 | 10.8±5.6 | 12.2±7.9 | 12.7±6.1 | 7.4±3.6 | 6.3±3.2 | 9.4±4.6 |
| *Botryotinia* | - | - | 0.6±0.5 | - | - | - | - | - | - | - | - | - |
| *Botrytis* | - | - | - | - | - | - | - | 0.7±0.6 | - | - | - | - |
| *Camarosporium* | 0.5±0.2 | 0.5±0.3 | 1.3±0.8 | - | - | - | 0.6±0.4 | 0.7±0.4 | 0.6±0.3 | 0.5±0.3 | - | - |
| *Cephaliophora* | 0.9±0.6 | 0.9±0.5 | - | - | - | - | - | - | - | - | - | - |
| *Chaetomium* | 0.9±0.5 | 0.9±0.6 | - | - | - | - | - | - | - | - | - | - |
| *Chalastospora* | - | - | - | - | - | 0.8±0.4 | - | - | 1.1±0.5 | 0.3±0.1 | 0.9±0-4 | 0.7±0.3 |
| *Chromelosporium* | 1.6±0.4 | 11.4±4.5 | 0.5±0.3 | - | - | 0.8± | 11.5±6.4 | 3.4±1.5 | 14.9±7.2 | 1.3±0.5 | 3.2±1.1 | 2.9±1.4 |
| *Cladosporium* | 12.7±5.3 | 12.7±5.5 | 13.0±5.3 | 21.5±7.3 | 31.8±10.4 | 14.7±3.24 | 0.6±0.3 | 1.4±0.3 | 3.3±1.5 | 1.5±0.7 | 3.5±1.2 | 4.0±2.1 |
| *Colletotrichum* | - | - | - | - | - | - | - | 0.7±0.5 | - | 0.3±0.2 | - | - |
| *Coniothyrium* | - | - | 1.2±0.8 | - | 3.4±2.1 | 1.1±0.9 | - | - | - | - | - | - |
| *Coniozyma* | 7.0±3.2 | 7.0±4.2 | 6.6±2.2 | 1.7±0.9 | 1.7±0.8 | 5.5±2.1 | 1.9±0.4 | 1.4±-0.3 | 1.7±0.4 | 1.3±0.6 | 0.3±-0.1 | 0.4±0.2 |
| *Coprinopsis* | - | - | 0.4 | 1.1±0.5 | - | - | - | - | - | - | - | - |
| *Cordyceps* | - | - | 0.2 | - | - | - | - | - | - | - | - | - |
| *Cosmospora* | 0.6±0.3 | 0.6±0.3 | - | - | 4.2±2.7 | 6.0±4.6 | - | 0.7±0.4 | - | 0.3±0.1 | 0.6±0.3 | 1.1±0.5 |
| *Curreya* | - | - | - | - | - | - | - | - | - | - | - | - |
| *Dendrothyrium* | - | - | 0.7±0.4 | - | - | - | - | 0.7±0.3 | - | - | 0.3±0.1 | 1.3±0.8 |
| *Derechslera* | - | - | - | - | - | - | - | - | - | - | - | - |
| *Diaporthe* | 0.2±0.1 | 0.2±0.1 | - | - | - | - | 2.5±1.4 | - | - | - | - | - |
| *Discosia* | - | - | 0.3±0.2 | - | - | - | - | 0.7±0.5 | - | - | - | - |
| *Dothiorella* | 0.9±0.3 | 0.9±0.4 | 0.8±0.2 | 1.0±0.8 | - | - | 0.6±0.2 | 0.7±0.4 | - | - | - | - |
| *Endoconidioma* | 2.3±1.2 | 2.3±1.2 | - | 1.3±0.9 | 2.5±1.3 | - | - | - | - | 0.3±0.1 | 1.4±1.0 | 1.6±1.3 |
| *Epicoccum* | 3.0±1.2 | 3.0±1.5 | 3.3±1.6 | 1.8±0.3 | 1.8±0.4 | 1.6±0.5 | 1.9±1.2 | 2.7±1.3 | 0.6±0.4 | 2.3±1.5 | 1.7±1.2 | 0.7±0.3 |
| *Eutypa* | - | - | - | - | - | - | - | - | 0.6±0.4 | - | - | - |
| *Fimetariella* | 0.4±0.2 | 0.4±0.2 | 0.2±0.1 | - | - | - | - | 0.7±0.3 | 1.7±0.6 | 1.0±0.6 | 0.3±0.2 | 0.7±0.2 |
| *Unknown* | - | - | 0.6±0.3 | - | - | - | - | - | 0.6±0.3 | - | 1.4±0.9 | 0.7±0.3 |
| *Fusarium* | 10.0±5.6 | 10.0±5.3 | 12.9±6.7 | 42.7±25.4 | 6.8±3.4 | 28.2±11.3 | 9.6±7.9 | 14.2±6.7 | 15.5±8.8 | 49.2±22.5 | 27.1±13.7 | 30.7±15.2 |
| *Gibberella* | - | - | - | - | 2.8±1.3 | - | - | 0.7±0.3 | 0.6±0.2 | - | 0.3±0.1 | - |
| *Heydenia* | 1.2±0.8 | 1.2±0.8 | 2.9±1.6 | - | 1.9±1.1 | - | 14.0±5.5 | 4.7±3.2 | 5.0±2.4 | 1.3±0.9 | 2.3±1.1 | 0.2±0.1 |
| *Hyalodendriella* | 5.8±2.6 | 5.8±2.5 | 5.1±3.1 | 1.5±0.8 | - | 1.2±0.3 | 2.5±1.2 | 3.4±1.4 | 0.6±0.3 | 1.5±0.9 | 0.3±0.1 | 2.5±1.3 |
| *Masoniella* | 0.2±0.1 | 0.2±0.1 | - | - | - | - | - | - | - | - | - | - |
| *Microsphaeropsis* | - | - | 1.8±1.5 | - | - | - | - | - | 0.6±0.5 | - | - | - |
| *Mollisia* | - | - | - | - | - | - | - | - | - | - | - | - |
| *Nectria* | - | - | - | - | - | 0.8±0.3 | - | - | - | - | - | - |
| *Neofabraea* | 2.4±0.8 | 2.4±1.2 | 2.2±1.1 | 0.9±0.3 | - | 3.3±1.6 | - | 0.7±0.5 | 1.1±0.5 | 1.3±0.9 | 3.5±2.1 | 1.1±0.6 |
| *Neofabraekienholzii* | - | - | 0.6±0.5 | - | - | - | - | - | - | - | - | - |
| *Ochrocladosporium* | 1.0±0.4 | 1.0±0.5 | 0.8±0.4 | - | - | - | 0.6±0.2 | - | - | - | - | - |
| *Penicillium* | 5.9±3.2 | 5.9± | 5.3± | 5.5±2.9 | 14.1±7.1 | 5.4±2.4 | 1.3±0.8 | 1.4±0.7 | - | 0.5±0.3 | 2.6±1.5 | 0.2±0.1 |
| *Pestalotiopsis* | - | - | - | - | - | - | - | 0.7±0.4 | 0.6±0.3 | - | - | - |
| *Phaeomoniella* | 2.4±1.5 | 2.4±1.8 | 3.6±2.5 | - | - | - | - | 2.0±1.1 | 2.2±1.2 | - | - | - |
| *Phaeosphaeria* | 3.9±2.8 | 3.9±2.8 | 3.2± | 1.2±0.8 | - | 3.1±1.6 | 2.5±1.4 | 5.4±3.4 | 3.9± | 2.0±1.0 | 2.3±1.6 | 7.2±5.6 |
| *Phaeosphaeriopsis* | 0.4±0.2 | 0.4±0.2 | - | - | - | - | - | - | - | - | - | - |
| *Phoma* | 6.1±3.2 | 6.1±3.4 | 5.5±3.6 | 7.5±3.6 | 15.3±8.8 | 1.1±0.3 | - | - | 1.7±0.8 | 0.3±0.1 | 3.7±1.5 | 0.4±0.2 |
| *Pithomyces* | 0.2±0.1 | 0.2±0.1 | - | - | - | - | - | - | - | - | - | - |
| *Plectania* | - | - | - | - | - | - | 5.7±3.6 | 0.7±0.5 | 0.6±0.4 | - | - | - |
| *Plenodomus* | - | - | - | 1.4±1.0 | - | - | - | - | - | - | - | - |
| *Porostereum* | - | - | - | - | - | - | - | - | - | - | - | - |
| *Prosthemium* | - | - | - | - | - | - | - | - | - | 0.3±0.2 | 2.0±1.6 | - |
| *Pseudocamosprium* | - | - | 0.4±0.3 | - | - | - | - | - | - | - | - | - |
| *Pseudocercospora* | 1.3±0.7 | 1.3±0.6 | 0.6±0.2 | - | - | 4.2±1.5 | 0.6±0.2 | 0.7±0.4 | - | 2.5±1.7 | 5.2±2.6 | 3.1±1.5 |
| *Pseudophaeomoniella* | - | - | 0.4±0.2 | - | - | - | - | - | - | - | - | - |
| *Purpureocillium* | - | - | - | - | - | - | - | - | - | - | - | - |
| *Pycnidiophora* | - | - | - | - | - | - | - | - | - | - | - | - |
| *Pyrenochaeta* | 0.5±0.2 | 0.5±0.2 | 0.9±0.3 | - | - | 1.1±0.6 | 0.6±0.3 | - | 0.6±0.2 | 1.3±0.7 | 1.2±0.4 | 0.7±0.4 |
| *Pyronema* | 2.6±0.8 | 2.6±0.9 | 1.7±0.8 | - | - | 1.3±0.7 | 7.6±3.4 | 18.9±9.1 | 2.8±1.0 | 1.0±0.5 | - | 0.2±0.1 |
| *Qualambaria* | 0.7±0.5 | 0.7±0.5 | - | - | - | - | - | - | - | - | - | - |
| *Rhinocladiella* | 0.8±0.6 | 0.8±0.6 | - | - | - | - | - | - | - | - | 1.2±0.5 | - |
| *Sarocladium* | 0.5±0.2 | 0.5±0.4 | - | - | - | - | - | - | - | - | - | - |
| *Sclerotinia* | - | - | 0.4±0.3 | - | - | - | - | - | - | - | - | - |
| *Scopulariopsis* | - | - | - | - | - | 1.6±1.4 | - | - | - | - | - | - |
| *Septoria* | - | - | - | - | - | - | - | - | - | 0.8±0.5 | 0.6±0.2 | 1.1±0.3 |
| *Sordaria* | 0.2±0.1 | 0.2±0.1 | 1.0±0.3 | 0.7±0.2 | - | 3.1±1.4 | - | 0.7±0.4 | - | 0.5±0.3 | 0.9±0.3 | - |
| *Spegazzinia* | - | - | 0.4±0.3 | - | - | - | - | - | - | - | - | - |
| *Stereum* | 0.3±0.2 | 0.3±0.2 | 0.4±0.3 | - | - | - | - | - | - | 1.0±0.7 | - | - |
| *Trametes* | - | - | 0.2±0.1 | - | - | - | - | - | 0.6±0.5 | - | - | - |
| *Tricharina* | - | - | 0.6±0.4 | - | - | - | 7.6±4.2 | 0.7±0.5 | - | 0.5±0.3 | - | 0.2±0.1 |
| *Trichoderma* | 0.6±0.4 | 0.6±0.4 | 0.7±0.5 | - | - | 0.4±0.2 | - | - | - | - | - | - |
| *Tumularia* | - | - | - | - | - | - | - | - | - | - | 0.3±0.2 | - |
| *Ulocladium* | - | - | - | - | - | - | - | - | 0.6±0.4 | 1.0±0.8 | 0.3±0.2 | - |
| *Ustilago* | 0.9±0.7 | 0.9±0.8 | - | - | - | - | - | - | - | - | - | - |
| *Valsaria* | 2.4±2.1 | 2.4±1.8 | 0.8±0.5 | 1.4±1.0 | - | - | - | - | - | - | - | - |
| *Verticillium* | 0.2±0.1 | 0.2±0.1 | - | - | - | - | - | - | - | - | - | - |
| *Xylaria* | - | - | - | - | - | - | - | 3.4±2.1 | - | 0.5±0.3 | - | 0.2±0.1 |

**Table S2** - ANOVA analysis to test significant differences (*P*-value) between fungal community groups obtained in the Canonical Correlation Analysis (CCA). Variation partitioning (*Varpart*) was calculated to achieve the total variance explained by each factor. *P*-values in bold are significant.

| Factors | *P*-value | *Varpart* (%) |
| --- | --- | --- |
| Total cultivar | **0.005** | **0.5%** |
| Total twig statuses | **0.005** | **3.9%** |
| Total fungal community | **0.005** | **5.2%** |
| Endophytic cultivar | **0.005** | **1.1%** |
| Endophytic twig statuses | **0.005** | **5.6%** |
| Epiphytic cultivar | **0.025** | **1.3%** |
| Epiphytic twig statuses | **0.005** | **5.9%** |
| Asymptomatic Total Cultivar | **0.049** | **1.5%** |
| Symptomatic Total Cultivar | **0.005** | **2.8%** |
| Asymptomatic Endophytic Cultivar | **0.010** | **-** |
| Symptomatic Endophytic Cultivar | **0.005** | **-** |
| Asymptomatic Epiphytic Cultivar | 0.050 | **-** |
| Symptomatic Epiphytic Cultivar | **0.005** | **-** |

**Table S3** - Analysis of similarity (ANOSIM), based on Bray-Curtis distance, between fungal communities inhabiting asymptomatic and OK-symptomatic twigs. R-statistics (R) and *P*-values of each variable are included.

| Variables | **ANOSIM** | |
| --- | --- | --- |
|  | R | *P*-value |
| Endophytic | 0.221 | 0.001 |
| Endophytic *Cobrançosa* | 0.246 | 0.001 |
| Endophytic *Madural* | 0.232 | 0.006 |
| Endophytic *Verdeal Transmontana* | 0.237 | 0.001 |
|  |  |  |
| Epiphytic | 0.239 | 0.000 |
| Epiphytic *Cobrançosa* | 0.498 | 0.001 |
| Epiphytic *Madural* | 0.211 | 0.020 |
| Epiphytic *Verdeal Transmontana* | 0.136 | 0.005 |
|  |  |  |
| *Cobrançosa* | 0.222 | 0.001 |
| *Madural* | 0.160 | 0.002 |
| *Verdeal Transmontana* | 0.009 | 0.001 |

**Table S4** – Functional categories of the several fungal operational taxonomic unit (OTU) identified in this work.

| **Fungal OTUs** | **Functional categories** |
| --- | --- |
| *Alternaria alternaria* | Plant pathogen/Others |
| *Alternaria alternata* | Plant pathogen/ Commensalistic |
| *Alternaria sp.2* | Plant pathogen/Others |
| *Alternaria brassicae* | Plant pathogen/ Commensalistic |
| *Alternaria infectoria* | Plant pathogen |
| *Alternaria metachromatica* | Pplant pathogen |
| *Alternaria multiformis* | Plant pathogen/Others |
| *Alternaria oregonensis* | Plant pathogen/Others |
| *Alternaria sp.3* | Plant pathogen/Others |
| *Alternaria solani* | Plant pathogen |
| *Alternaria sp.1* | Plant pathogen/Others |
| *Alternaria sp.4* | Plant pathogen/Others |
| *Alternaria tenuissima* | Plant pathogen/Others |
| *Anthostomella leucospermi* | Unknown |
| *Arthrinium arundinis* | Plant pathogen |
| *Arthrinium phaeospermum* | Plant pathogen |
| *Arthrinium sp.* | Plant pathogen |
| *Ascobolus crenulatus* | Unknown |
| *Ascobolus sp.* | Unknown |
| *Aspergillus brasilienses* | Plant pathogen |
| *Aspergillus felis* | Plant pthogen |
| *Aspergillus sp.* | Plant pathogen |
| *Aspergillus sydowii* | Plant pathogen |
| *Aspergillus tennesseensis* | Plant pathogen |
| *Aspergillus versicolor* | Plant pathogen |
| *Aureobasidium pullulans* | Beneficial |
| *Aureobasidium sp.* | Beneficial |
| *Beauveria bassiana* | Plant pathogen |
| *Biscogniauxia mediterranea* | Plant pathogen |
| *Botryotinia fuckeliana* | Plant pathogen |
| *Botrytis cinerea* | Plant pathogen |
| *Camarosporium aloes* | Unknown |
| *Camarosporium brabeji* | Unknown |
| *Camarosporium leucadendri* | Unknown |
| *Camarosporium psoraleae* | Unknown |
| *Camarosporium sp.* | Plant pathogen |
| *Cephaliophora tropica* | Unknown |
| *Chaetomium elatum* | Plant pathogen |
| *Chaetomium sp.* | Plant pathogen/Others |
| *Chalastospora gossypii* | Unknown |
| *Chromelosporium carneum* | Unknown |
| *Cladosporium allicinum* | Unknown |
| *Cladosporium cladosporioides* | Plant pathogen/Beneficial |
| *Cladosporium herbarum* | Plant pathogen/ Commensalistic |
| *Cladosporium sp.* | Plant pathogen/ Commensalistic |
| *Colletotrichum dematium* | Plant pathogen |
| *Colletotrichum sp.* | Plant pathogen |
| *Comospora sp2.* | Unknown |
| *Coniothyrium palmicola* | Unknown |
| *Coniothyrium sp.* | Plant pathogen/ Beneficial/ Commensalistic |
| *Coniozyma leucospermi* | Unknown |
| *Coprinellus aff. radians* | Others |
| *Coprinellus angulatus* | Others |
| *Coprinellus sp.* | Others |
| *Coprinellus xanthothrix* | Unknown |
| *Coprinopsis candidolanata* | Others |
| *Coprinopsis cinerea* | Others |
| *Cordyceps confragosa* | Unknown |
| *Cosmospora sp.* | Unknown |
| *Cosmospora sp.1* | Unknown |
| *Croprinopsis sp.* | Others |
| *Curreya grandicipis* | Unknown |
| *Cytospora eucalyptica* | Unknown |
| *Dendrothyrium variisporum* | Unknown |
| *Derechslera triseptata* | Plant pathogen |
| *Diaporthe ambigua* | Plant pathogen |
| *Diaporthe rudis* | Plant pathogen |
| *Discosia sp.* | Plant pathogen |
| *Dothiorella iberica* | Plant pathogen |
| *Endoconidioma populi* | Unknown |
| *Epicoccum nigrum* | Plant pathogen/ Beneficial |
| *Eurotium rubrum* | Unknown |
| *Eutypa tetragona* | Plant pathogen |
| *Fimetariella rabenhorstii* | Unknown |
| *Fungal endophyte sp24.* | Unknown |
| *Fungal endophyte sp27.* | Unknown |
| *Fungal endophyte sp30.* | Unknown |
| *Fungal epiphyte sp.17* | Unknown |
| *Fungal epiphyte sp6.* | Unknown |
| *Fusarium solani* | Plant pathogen |
| *Fusarium avenaceum* | Plant pathogen |
| *Fusarium globosum* | Plant pathogen |
| *Fusarium lateritium* | Plant pathogen |
| *Fusarium oxysporum* | Plant pathogen |
| *Fusarium proliferatum* | Plant pathogen |
| *Fusarium sp.* | Plant pathogen / Commensalistic |
| *Fusarium sp2.* | Plant pathogen |
| *Fusarium tricinctum* | Plant pathogen |
| *Fusarium verticillioides* | Plant pathogen |
| *Gibberella avenacea* | Plant pathogen |
| *Gibberella fujikuroi* | Plant pathogen |
| *Glomerella sp.* | Plant pathogen |
| *Heydenia alpina* | Unknown |
| *Heydenia sp.* | Unknown |
| *Hyalodendriella betulae* | Unknown |
| *Lecythophora hoffmannii* | Plant pathogen/ Beneficial |
| *Malbranchea cinnamomea* | Unknown |
| *Masoniella sp.* | Unknown |
| *Microsphaeropsis olivacea* | Plant pathogen/ Beneficial/ Others |
| *Microsphaeropsis proteae* | Unknown |
| *Mollisia minutella* | Unknown |
| *Nectria illudens* | Others |
| *Neofabrae alba* | Plant pathogen |
| *Neofabrae sp.* | Plant pathogen |
| *Neofabraea malicorticis* | Plant pathogen |
| *Neofabraekienholzii* | Unknown |
| *Ochrocladosporium adansoniae* | Unknown |
| *Ochrocladosporium sp.* | Unknown |
| *Paraconiothyrium brasiliense* | Beneficial |
| *Penicillium brevicompactum* | Unknown |
| *Penicillium canescens* | Plant pathogen |
| *Penicillium cecidicola* | Unknown |
| *Penicillium corylophylum* | Plant pathogen |
| *Penicillium cyclopium* | Beneficial |
| *Penicillium echinulatum* | Unknown |
| *Penicillium expansum* | Plant pathogen |
| *Penicillium glabrum* | Plant pathogen |
| *Penicillium polonicum* | Plant pathogen |
| *Penicillium rugulosum* | Unknown |
| *Penicillium sanguifluum* | Unknown |
| *Penicillium sp.* | Plant pathogen/ Beneficial |
| *Penicillium sp3* | Plant pathogen/ Beneficial |
| *Penicillium spinulosum* | Unknown |
| *Pestalotiopsis australis* | Unknown |
| *Pestalotiopsis caudata* | Unknown |
| *Phaeomoniella sp.* | Plant pathogen |
| *Phaeomoniella sp.1* | Plant pathogen |
| *Phaeomoniella sp2* | Plant pathogen |
| *Phaeosphaeria avenaria* | Plant pathogen |
| *Phaeosphaeria sp.* | Plant pathogen |
| *Phaeosphaeria sp.1* | Plant pathogen |
| *Phaeosphaeria sp.9* | Plant pathogen |
| *Phaeosphaeriopsis glaucopunctata* | Plant pathogen |
| *Phoma aloes* | Unknown |
| *Fungal epiphyte sp.3* | Unknown |
| *Fungal epiphyte sp.4* | Unknown |
| *Phoma sp.* | Plant pathogen/ Others |
| *Phoma sp.1* | Plant pathogen/ Others |
| *Pithomyces chartarum* | Plant pathogen |
| *Plectania rhytidia* | Unknown |
| *Plenodomus enteroleucus* | Unknown |
| *Porostereum spadiceum* | Unknown |
| *Pseudocamosprium sp.* | Unknown |
| *Pseudocercospora cladosporioides* | Plant pathogen |
| *Pseudocercospora sp.* | Plant pathogen |
| *Pseudophaeomoniella oleicola* | Unknown |
| *Purpureocillium lilacinum* | Others |
| *Pycnidiophora aurantiaca* | Unknown |
| *Pyrenochaeta corni* | Unknown |
| *Pyrenochaeta sp.* | Plant pathogen |
| *Pyrenochaeta sp1.* | Unknown |
| *Pyronema domesticum* | Unknown |
| *Qualambaria sp.* | Plant pathogen |
| *Rhinocladiella similis* | Unknown |
| *Sarocladium strictum* | Plant pathogen/ Others |
| *Sclerotinia sclerotiorum* | Plant pathogen |
| *Scopulariopsis brevicaulis* | Others |
| *Septoria eucalyptorum* | Unknown |
| *Septoria sp.* | Plant pathogen |
| *Sordaria fimicola* | Beneficial |
| *Sordaria macrospora* | Plant Pathogen |
| *Sordaria sp.* | Plant Pathogen |
| *Fungal epiphyte Sp18* | Unknown |
| *Spegazzinia tessarthra* | Unknown |
| *Stereum annosum* | Unknown |
| *Trametes sp1.* | Unknown |
| *Trametes sp2.* | Unknown |
| *Tricharina praecox* | Unknown |
| *Tricharina sp.* | Unknown |
| *Tricharina striispora* | Unknown |
| *Trichoderma sp.* | Beneficial |
| *Ulocladium sp.* | Plant pathogen |
| *Ustilago bullata* | Plant pathogen |
| *Valsaria insitiva* | Plant pathogen |
| *Valsaria sp.* | Plant pathogen |
| *Valsaria spartii* | Unknown |
| *Verticillium nigrescens* | Plant pathogen |
| *Xylaria arbuscula* | Others |
| *Xylaria sp1.* | Others |
